# Correspondence

**Published:** 1868-09

**Authors:** F. K. Bailey

**Affiliations:** Knoxville, Tenn.


### Correspondence.

KNOXVILLE, Tenn., July, 1868.

PROF. N. S. DAVIS:

The readers of the EXAMINER may be interested in a brief account of this region of country, lying between the Cumberland and Smoky Mountains. This valley is about 80 miles in width, and 1000, or more, feet above the level of the sea. The Holston river has its origin in South-west Virginia, and, running about midway of the country between the two mountain ranges, unites with other streams to form the Tennessee. This whole region is hilly, but the soil is capable of being tilled to the summits of the elevations. Knoxville is built upon three hills, on the north bank of the river, and between the elevations run two creeks. The drainage is, consequently, most excellent, and but little water can become stagnant.

From the first settlement of this country, in 1792, until 1834, intermittents were said to have been unknown, except upon the river bottoms. From 1834, this form of disease began to prevail, and culminated, in 1838, in an epidemical form, which proved terribly severe and fatal. From a history of this epidemic, before me, it seems to have been of a type similar to that which was so prevalent the same year in many newly settled localities in the North-western States. "Black vomit occurred in some cases, and many considered themselves in the midst of yellow fever." Since that time, no general epidemic has prevailed to any extent.

From an observation of about ten months, there is every reason to believe that this country is as healthy as Eastern New York, or New England. The weather in winter is mild, the mercury seldom going down to zero. Snow seldom falls more than three inches deep, and melts off in a few days. There being no marshes, paludal influences are unknown. A case of ague is scarcely known. The fevers are generally simple in their character, and remittent in their type. Last winter a great many cases of neuralgia occurred, principally located in the supraorbital region, and generally over one eye; the pain was most severe about noon, and subsided towards night; there was no chill on its accession, and but little fever. Mild alteratives, followed by tonics, soon removed the trouble.

Pneumonia prevailed in February and March, but confined principally to the negroes. From the want of proper shelter and nursing, this affection proved fatal in many instances. The blacks are peculiarly liable to pulmonary disease, and the mulattoes are more so than the full-blooded. Scrofula is often met with, but seems confined to families where defective nutrition, resulting from poverty, is the most obvious cause. The class known as "poor whites" are most affected. Phthisis pulmonalis is not often seen, except in cases where the disease is excited by hereditary influences. Asthma is seldom met with, and those who have been afflicted with that distressing disease find marked relief after a short residence here.

The operation of entire removal of the uterus was performed, some years ago, in this place, by the late Dr. Baker. The subject was a negress, laboring for some time under what was considered ovarian disease, and the operation was commenced for the purpose of removing the diseased organ. On proceeding, after the first incision was made, the entire uterus was found involved, and the operator concluded he would extirpate the whole mass. Recovery was rapid, and the woman is still living here in good health. The morbid specimen is still preserved, and in possession of Dr. Boyd, a student of Dr. Baker.

Dr. James Rodgers, of this city, informs me that he himself removed the uterus, in the case of a negress. The tumor was adherent to the rectum, and an opening had formed between the two organs. The removal of the uterus, consequently, left an opening still in the rectum, which was closed by a stitch, with the hope that union might take place. The case went on favorably for five days, and the external incision had partially united by the first intention, when, from a motion from the bowels occurring, the stitch was torn out, the contents escaped into the abdominal cavity, and death occurred as in perforation.

The Tennessee Asylum for the Education of Deaf Mutes is located here, and has, at present, about 50 inmates. The location is a fine one, and the buildings and grounds are in excellent condition.

Some benevolent person donated, a few years ago, a sum of money, for the purpose of establishing a hospital at this place. There is a large lot and some $2000 or $3000 in money now available; and when the State finances will admit, no doubt there will be means appropriated for placing the institution in a way of practical operation.

There are numerous mineral springs in different localities in East Tennessee, and at some points there are accommodations for a large number of visitors.

Previous to the war, the East Tennessee Medical Society was in successful operation. By special charter it had the right to grant diplomas, and included most of the medical men in the whole region. This Society has not been revived since the war, but it is contemplated to take measures at an early day to accomplish its reörganization. F. K. BAILEY.

